# Society Meetings

**Published:** 1901-06

**Authors:** 


### SOCIETY MEETINGS.

THE American Medico-Psychological Association will hold its 57th annual meeting at the Hotel Pfister, Milwaukee, June 11-14, 1901. Dr. P. M. Wise is the president, and Dr. C. B. Burr is the secretary.

THE American Medical Editors' Association will hold its annual meeting at St. Paul, June 3, 1901, under the presidency of Dr. Alex. J. Stone.

THE National Association for the Study of Epilepsy and the Care and Treatment of Epileptics held a meeting at Washington, May 14 and 15, 1901, under the presidency of William P. Letchworth, LL.D. Dr. Wm. P. Spratling is the secretary.

THE Buffalo Academy of Medicine held meetings during the month of May, 1901, as follows:

*Section on Surgery.*—Tuesday evening, May 7th. Program: Something new regarding stricture, J. Henry Dowd.

*Section on Medicine.*—Tuesday evening, May 14, 1901. Program: The relation of the medical profession in the twentieth century to the tuberculosis problem, S. A. Knopf, New York; The paper was discussed by Drs. Charles G. Stockton, Ernest Wende, John Pryor, DeLancey Rochester and B. G. Long.

*Section on Ophthalmology, Otology and Laryngology.*—Monday evening, May 20th. Program: Exhibition of an instrument for measuring the balance of ocular muscles, Elmer E. Starr; Sinus phlebitis and thrombosis, John E. Sheppard, Brooklyn, N.Y.

*Section on Pathology.*—Tuesday evening, May 21st. Program: Original researches on trichinosis—illustrated—Herbert U. Williams; The brachial plexus, Abram T. Kerr.

*Section on Obstetrics.*—Tuesday evening, May 28th. Program: Excision of the bladder, Matthew D. Mann.

The American Dermatological Association holds its 25th annual meeting at the Beach Hotel, Chicago, May 30 and 31, and June 1, 1901.

---

The Medical Society of the County of Erie will hold its 80th semi-annual meeting at the Y. M. C. A. parlors, corner of Mohawk and Pearl Streets, Tuesday, June 11, 1901, beginning at 10 o'clock a.m., under the presidency of Dr. Wm. C. Phelps. Dr. Henry L. Elsner, of Syracuse, President of the Medical Society of the State of New York, will read a paper and Dr. Henry R. Hopkins, of Buffalo, will present the subject of schoolhouse sanitation. A full attendance is desired.

---

The Mississippi Valley Medical Association has changed the date of its meeting to the 12th, 13th and 14th of September, 1901. It is to be held at Put-in-Bay Island (Lake Erie), O.

